# Emasculation of Masturbators—Is It Justifiable?

**Published:** 1894-11

**Authors:** 


### Abstracts and Selections.

### Emasculation of Masturbators.—Is It Justifiable?

A most interesting and sensational controversy has been for some time going on in Kansas between the newspapers and the *Kansas Medical Journal*, growing out of the action of Dr. F. Hoyt Pilcher's action in castrating a number of boys confirmed in the evil habit of masturbating,—inmates of the Asylum for Idiots and Feeble-Minded Youths,—of which institution Dr. P. is superintendent. Dr. Pilcher was bitterly denounced by certain newspapers, and the action characterized as "cruel," "brutal" and "unjustifiable." The Doctor says this is political campaign thunder. The several publications on the subject are interesting; they are important, too, in the respect, that they show two things: 1st, that there is a growing sentiment in the profession in favor of castration, not only for disease, but as a prophylactic against a long train of evils, and particularly against the hereditary transmission of vice, disease, and the propensity to crime; and 2d, that the public are not prepared for anything of the kind and must be educated up to it. Much literature, and some of it very good, has appeared upon the subject in medical journals, and the conviction that sooner or later emasculation must be substituted for capital punishment, as well as made a penalty for certain minor offenses, is rapidly gaining ground.

The JOURNAL reproduces the following as showing this tendency. The following is from the *Kansas Farmer*, under the head of "The Family Doctor," a department edited by Dr. Henry Roby, of Topeka :

### THE WINFIELD HORROR.

FAMILY DOCTOR:—Will you kindly tell us whether the operation of Dr. Pilcher on the boys in the asylum at Winfleld was humane and justifiable, or whether it was, as some people claim, a brutal crime and deserving of the penitentiary? E. B. C.

The presumption of both law and science is in favor of the doctor. I have no personal acqnaintance with him and no knowledge of his skill and judgment, no knowledge of the conditions of these particular boys, but the presumption is in favor of the treatment said to have been administered. Emasculation is not a crime when done to save a life, or to cure an insanity or an imbecility, as it often is. It is no crime when it is done to restrain a diseased boy from an otherwise incurable tendeucy to self-destruction, either of suicide or the sure damnation of an unchecked secret vice.

Castrates are very common in oriental countries. Herodotus says that in Persia they were especially prized for fidelity and honor. Justinian's most famous general was an eunuch. The histories of China, India, Persia, Greece and Rome abound in instances of emasculation for various purposes aside from curing disease. From time immemorial to the days of Pope Leo XIII, the vatican choir was supplied with male soprano singers made such by castration. That custom is still very prevalent in Mohammedan countries. All the harems and seraglios are guarded by eunuchs. Many people all over the world emasculate themselves as a religious rite. That large and religious sect in Russia, called Skopjis, are all eunuchs. In the third century the celebrated Origen instituted at Alexandria a flourishing sect on that line, taking his cue from Matthew xix, 12.

There is no just ground of belief that the generative glands in the human species are any more exempt from disease and dangerous disorders than any of the other numerous glands, large and small, in the human economy. They are all subject to disease and malformation, and all alike must be subjected at times to the mercies of surgery in order that life and intellect, or both, may be saved. These glands are no more sacred or infallible than the liver or spleen or kidneys or the brain. All these must fall under the surgeon's hand in many instances that life may be preserved and intellect maintained.

The sexual glands in both cases are the very common seat of serious disease. They are the very common source of insanity and crime when their physiological activities are perverted or reversed. Countless murders have been committed under the ardor and fury of a sexual pervert, and many nameless crimes and outrages in domestic life have their origin in disordered sex glands. Many suits for divorce in our courts would be avoided if all the people had perfectly normal reproductive glands. Thousands of invalid women have been restored to health by the removal of hopelessly diseased ovaries.

I am quite sure that if given the authority of law I could go through the jails, asylums and prisons of this country and emasculate a large percentage of the inmates and turn them out in perfect safety to society, where now it would be the extreme of danger and folly to relax their strict and grim restraint. Just as the fury of the war horse is taken out of him by gelding, so the fury of a criminal is. It would be a most beneficent thing for society and the social compact called the state, if all criminals were emasculated. The impulse to crime would be very largely eliminated thereby and the reproduction of criminals would be stopped. Who can measure in money or in words the incalculable benefit to the race if all criminals of both sexes were thus prevented from spawning their ilk in the great stream of human life? Criminal? Nay! Nay! A God's blessing to the world, a boon to all mankind. Who keeps up the steady stream of criminals from age to age? Criminal progenitors largely. Who educate the innocent and unguarded in the ways of crime? The offsprings of crime who ought to have been rendered incapable of reproduction.

Society—the State—has the power to protect itself against the fearful prevalence of ravaging crime by any well-timed and well-adapted and humane means that wisdom can devise. But this, the most beneficent, humane and successful of all forms of defensive restraint, is left unused, because, forsooth, like false modesty, a false sense of glandular sacredness stands in maudlin mockery and scoffs at the right.

How many rapes, think you, would be committed, if the penalty were certain and swift castration? Why not make the punishment fit the crime? Then, at least, they would never be repeated. This, the only reason—that from a very primitive age of the world, when mankind engaged in Phallic worship, that is worshiping their own generative organs as the creator, down to the present, a taint of that old worship still clings to the race, and one set of organs is esteemed too sacred for invasion even for the beneficent purposes of cure. A wiser doctrine is found in these words from the sacred writings: "If thine eye offend thee, pluck it out. If thy hand offend thee, cut it off and cast it from thee," etc. And if a procreative gland offend thee (seriously) pluck it out and cast it from thee, is equally good, wise and just doctrine. We do it with all other glands amenable to surgery, and why not this one, which is the railroad switch of human life, that derails many a train of fine human impulses from the track of virtue, sobriety and rectitude and plunges it into the ditch of debauchery and crime.

The next legislature can do the State no greater service than to provide for the elimination of uncounted generations of uncreated criminals who are otherwise sure to be a heavy burden on all right-minded and virtuous citizens, and also to provide proper authority and legal support for treating certain public charges according to the most advanced and humane methods of medicine and surgery.

---

### CASTRATION FROM A LEGAL STANDPOINT.

The agitation of the Winfield affair in the daily newspapers may result in great benefit, not in a political sense, but in enlightening the people upon a subject of much importance to them. Could the public be convinced that by removing the cause, out of imbecility might grow manhood, and out of debauchery and crime might grow honesty and integrity, there would be need of fewer such institutions as that at Winfield. The operation for the emasculation of the sexes loses all its horror when looked upon from a scientific standpoint. The ignorant, irrational or criminal application of any good measure will soon make it obnoxious to the public mind. The emasculation of the female for diseased conditions no longer meets with that disapproval which characterized the first period of its history. The operation upon the male possesses fully as many and as great advantag e as are accorded the other. The operation should never be sanctioned by the physician unless it is, in his opinion, the last resort for a cure.

It ought to be resorted to whenever, in the best judgment of the physician, in every case where it is the one remedial cure of a condition or disease that threatens the life or sanity of the person, and without which both would be in danger.

There never has been a thought on the part of the State of enacting laws regulating any particular practice of the physician in the honest discharge of his function. He has always done so untrammeled. The law has made it a crime for a physician, under the pretense of practising his profession, to commit abortion, and sends him to the penitentiary for so doing; but in all such laws there is a clause that declares it shall be no crime if the same was done, in the opinion of the physician, to save life. No State, by its laws, looks after an honorable physician in his practice. He is in every way protected. He can lop a limb, take out an eye, make an exsection, reduce the linear capacity of the intestines, amputate a hand, and he needs neither notify the police or call in the county attorney. The necessity and his intelligence and honor give him that right. And it is just the same with castration for any cause or reason.

Dr. Wilber's statement that there are laws existing to punish the physician who makes the operation, is incorrect. There is no such law on the statute books of the world. There could not be so long as reason possesses the legislature.

The physician is above the law, so long as his intelligence and knowledge keep abreast the state of the art in his profession, so long as he is honorable and does not lend himself as a tool to the vicious for the commission of crime.

The medical authority of a public institution is there to advise and is there to act in all cases where the disease or condition confronts him. And he can act just as any other honorable and intelligent physician might act in his private practice, with this suggestion: That our institutions are largely in the hands of politicians, and the physician who would expect fair treatment at their hands when it became necessary to do otherwise, is too ignorant of the ways of the world to be at the head of such an institution, and for their own protection it should be upon the pronounced advisement of eminent medical men, proceeded with dignity and done openly; but this is suggested for the personal protection of the physician from the political wolves of all parties, who would at once clamor for his scalp, if party interests were to be subserved thereby.

Speaking about this operation, there are some situations where the legislature should compel its performance.

A brute who has been guilty of the crime of forcible rape should be prevented from further contaminating our race, and in the neglect to do so, there will be trouble with such a fellow three hundred years after he is dead.

The incorrigible criminal, who yields to neither punishment or leniency, often serves his sentence and returns to society, and unless he is served in like manner, he will haunt the country with his crime generations after he is dead and forgotten.—*Kansas Medical Journal.*

---

### THE WINFIELD CASE.

A political supplement of the Topeka *Lance*, of September 1, 1894, contains eight columns or more denouncing certain operations of Dr. Pilcher at the asylum for idiotic and imbecile youth, established by the State at the city of Winfield. It appears from the face of the article that there were castrations of several boys who had been long confirmed in a vicious habit, which had not yielded to prior treatment, and which operations had been openly discussed and intelligently considered by Dr. Pilcher and other medical men, and which had been sanctioned and advised by a council of capable and efficient physicians. The whole trend of the article alluded to was not in the interest of the science of health or right living, but had for its one supreme object the political purpose to envenom the voter against the administration under whose reign these operations were accomplished. It is hardly considered the province of a medical journal to seek opportunities to reply to campaign documents, but when they involve the honor and standing of the medical profession, it is necessary as a matter of defense, and the ordinary tribute demanded of a professional man to his profession. It appears by the statement of Dr. Wiles, a former superintendent, that several of the persons operated on had in his time been inmates, had long been addicted to the vice, and notwithstanding a course of treatment by him, which he designates as moral suasion, and consisting of good fatherly advice mingled with the persuasiveness of a straight jacket, these persons still continued to be under complete dominion of that vice, and riveted more firmly to it by the emaciated body and enervated mind that are necessarily incident to long continued processes of that kind. The article clearly discloses that moral suasion as practiced by Dr. Wiles in such cases was absolutely ineffective, and cost the State the expense of constant attendance to simply prevent and not to cure. The testimony undisputed throughout this article is as it had to be, if they proposed to speak *ex cathedra*, that a surgical operation is a sure and lasting cure; that from the time of the operation the body, shorn of a diseased and brutal functions, recovers and gets strong; that the mind, if the lapse has not been too extreme, also recovers and resuscitates all there is of the man to be saved. Dr. Pilcher met this state of affairs when he took charge. He had the futile methods and experiences of Dr. Wiles before him. He could have continued the same treatment, and so could his successors, in the end leaving for the State, relatives and society, idiocy and total bestiality, but without mayhem. Dr. Wiles was in charge four years and eight months, and it is no unjust criticism to say with such poor results from his course of moral suasion, he ought to have changed the treatment, more straight jacket and probably less advice, or *vice versa*. One thing is certain: prevention was all he sought at a constant expense to the State, and with increasing disability and a stronger passion as the habit continued.

Dr. Pilcher, like a brave and capable man, sought something better. There could be much saved from such wrecks. He could give back a restored mind and robust health, a bestial function destroyed, and he did it. He called around him a council of competent medical men; they determined on the operations, for here was cure, and the operations were performed, and for which he should have the profound respect and acknowledgement of the State, humanity and kindred.

This political article has sought among physicians for anathema, and found Dr. Wilbur, of Kalamazoo, Mich., who declares that such operations are unlawful, unjustifiable, and physicians who counsel or practice it should be lodged in the penitentiary, and who declares it unlawful in most States. These operations are old as the profession, are the remedy, and only remedy, for extreme and rebrobate cases, recognized as legitimate in the profession, and constantly practiced by men eminent in standing and learning. If any State or Nation has declared it unlawful and punishable for the physician to make such an operation as a remedy for this devastating vice, and forbids him under the displeasure of its laws from so doing, we have not been informed of it. There may be, but Dr. Wilbur must not confound the punishment meted out to offenders who, for some malevolent purpose, commit this sort of mutilation on the possessor of a sound mind in a sound body.

These operations are occurring constantly under the trained eye of skilled physicians, and all honor to the devoted man who seeks cure and restoration and gives back to the State a restored citizen, to society an ornament instead of a burden, and who restores him to friends and family clothed in his right mind, robbed only of a beastly and execrated curse.

It is one thing to fling ordinary political mud, and it is another thing to denounce proper conduct and enlightened methods to accomplish the change of a few votes.—*Kansas Medical Journal*, September 8, 1894.

